# Extranodal Biphenotypic Non-Hodgkin Lymphoma of the Popliteal Cavity: A Case Report and Review of Literature

**DOI:** 10.3390/diagnostics12071649

**Published:** 2022-07-07

**Authors:** Giulia A. Restivo, Lara Mussolin, Paolo D’Angelo, Angelica Zin, Martina Pigazzi, Elisa Carraro, Emanuele S. G. D’Amore, Marta Pillon, Piero Farruggia

**Affiliations:** 1 Department of Health Promotion, Mother and Child Care, Internal Medicine and Medical Specialties “G. D’Alessandro”, University of Palermo, 90127 Palermo, Italy; 2 Clinic of Pediatric Hemato-Oncology, Department of Women’s and Children’s Health, University of Padova, 35128 Padova, Italy; lara.mussolin@unipd.it (L.M.); martina.pigazzi@unipd.it (M.P.); elisa.carraro87@gmail.com (E.C.); marta.pillon@unipd.it (M.P.); 3 Institute of Pediatric Research-Fondazione Città della Speranza, 35127 Padova, Italy; angelica.zin@unipd.it; 4 Department of Pediatric Hemato-Oncology, ARNAS Ospedali Civico, G. Di Cristina, 90127 Palermo, Italy; paolo.dangelo@arnascivico.it (P.D.); piero.farruggia@arnascivico.it (P.F.); 5 Department of Pathology, San Bortolo Hospital, 36100 Vicenza, Italy; emanuele.damore@tin.it

**Keywords:** extranodal lymphoma, soft-tissue lymphoma, biphenotypic lymphoblastic lymphoma, childhood

## Abstract

Primary soft-tissue lymphoma (PSTL) is a rare extranodal non-Hodgkin lymphoma, characterized by a mass growing within soft-tissue, which is connective tissue, adipose tissue, and skeletal muscle. Here, we describe a case of biphenotypic lymphoblastic lymphoma arising from soft tissue of the popliteal fossa in an 11-year-old boy. A pediatric review about PSTL revealed that anaplastic large cell lymphoma is the most common histological type and a biphenotypic lymphoblastic lymphoma has not yet been reported in childhood. Lymphoma should always be considered in patients presenting with a soft-tissue mass, and a comprehensive immunohistochemical evaluation, including B-cell, T-cell, and myeloid markers, is needed to make a correct diagnosis and establish the most suitable treatment.

## 1. Introduction

Primary extranodal non-Hodgkin lymphoma (PE-NHL) typically arises from tissue other than lymph nodes and even from sites that normally do not contain lymphoid tissue; the incidence rate ranges from 24% to 48% of all NHL [1,2]. A variety of PE-NHL is primary soft-tissue lymphoma (PSTL), representing about 0.1% of all malignant lymphomas [3]. It is characterized by a primary mass growing within soft-tissue, which includes connective tissue, adipose tissue, and skeletal muscle. This rare condition must be distinguished from the most frequent event in which soft-tissue is involved secondarily, as a consequence of direct spreading or hematogenous dissemination from a nodal or extranodal lymphoma [4]. The majority of PSTL occurs in adulthood, while children are exceptionally affected [5].

We describe a case of a pediatric PSTL localized in the popliteal fossa, with an outstanding histological characterization; a review about this topic was conducted thereafter.

## 2. Materials and Methods

Detailed immunophenotyping was performed on paraffin sections from tissue sample; the staining panel included CD2, CD3, CD4, CD5, CD7, CD8, CD10, CD11c, CD13, CD14, CD15, CD19, CD20, CD22, CD33, CD34, CD38, CD43, CD45, CD68, CD79a, CD99, CD117, CD163, myeloperoxidase (MPO), terminal deoxynucleotidyl transferase (TdT), cytoplasmic µ chains, vimentin, PAX5, FLI-1, lysozyme, Myf4, MND116, and EMA.

As regards the molecular cytogenetic screening for chromosomal abnormalities associated with myeloid and lymphoid leukemias, total RNA was isolated using Trizol (Invitrogen); one microgram of RNA was reverse-transcribed into cDNA using the SuperScript II system (Invitrogen) according to the manufacturer’s instructions. Reverse transcriptase-polymerase chain reaction (RT-PCR) was performed for the evaluation of all rearrangements.

A literature review about pediatric PSTL was conducted using PubMed, Scopus, and Web of Science, combining the terms “soft-tissue” AND “non-Hodgkin lymphoma”, “connective tissue” AND “non-Hodgkin lymphoma”, and “skeletal muscle” AND “non-Hodgkin lymphoma”; 12 cases were retrieved.

## 3. Case Presentation

**Figure 1:**
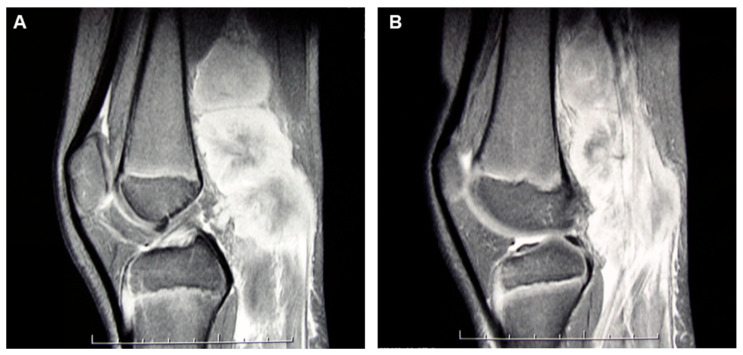
(**A**,**B**). MRI of the right knee revealed a 5 × 4 × 10 cm multinodular lesion localized in the distal region of the posterior thigh and in the popliteal fossa, isointense to muscle on T1-weighted images and with intermediate signal intensity on T2-weighted images, showing intense and not homogeneous contrast enhancement.

**Figure 2:**
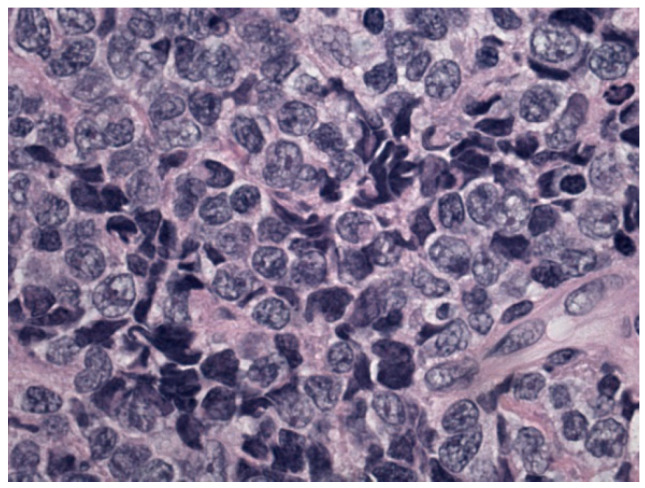
Morphological details investigated using hematoxylin and eosin staining: monomorphic population of blasts.

**Figure 3:**
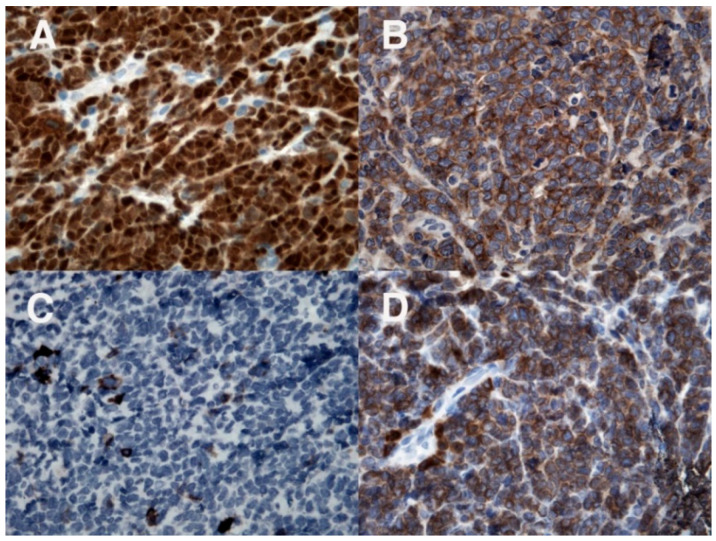
Immunohistochemical evaluation: positivity for TdT (**A**) and B-cell markers CD19 (**B**) and CD79a (**D**); CD20 (**C**) is negative.

**Figure 4:**
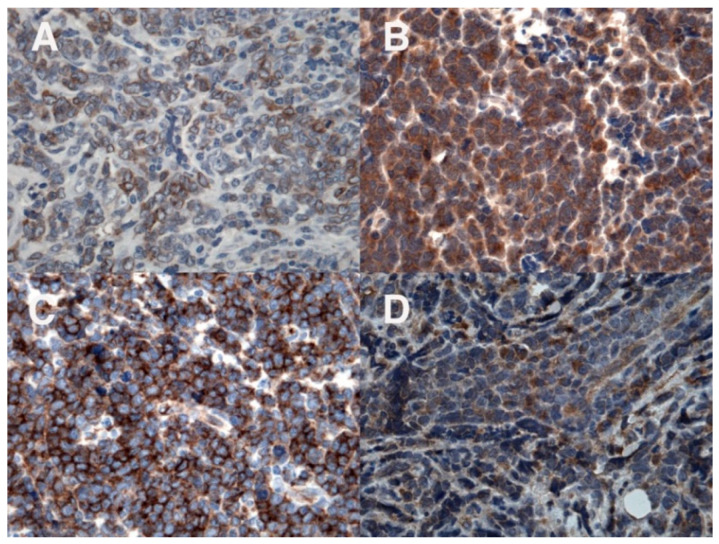
Immunohistochemical evaluation: positivity for the myeloid marker MPO (**A**); in addition, positivity for CD33 (**B**), CD34 (**C**), and CD117 (**D**).

An 11-year-old boy suffered from a 2-months history of a growing mass in his right popliteal fossa; he did not complain of any other symptoms. Due to progressive increase in size of the popliteal mass, a magnetic resonance imaging (MRI) of the right knee was performed; it revealed a 5 × 4 × 10 cm lesion, localized in the distal region of the posterior thigh and in the popliteal fossa, isointense to muscle on T1-weighted images and with intermediate signal intensity on T2-weighted images, showing intense and not homogeneous contrast enhancement; concomitant edema in subcutaneous adipose tissue and in gastrocnemius muscle fascia was detected (Figure 1A,B). The boy was admitted to another hospital; a needle biopsy was performed, which showed an undifferentiated malignant tumor with small round cells, positive for vimentin and CD99, negative for CD45, CD20, and CD3, suggesting initially the diagnosis of Ewing’s sarcoma/peripheral primitive neuroectodermal tumors (pPNET). Ten days after, the boy came to our attention. On physical examination, a painless, hard, and fixed mass was palpable in the posterior side of the right knee, with evident venous reticulum of the overlying skin, and a clear discrepancy of circumference between the two legs (29 cm in the right one versus 25 cm in the left one) was found; no enlarged lymph nodes were noticed. Blood examinations were all in normal range as well as bilateral bone marrow aspirates; a new incisional biopsy of the mass was performed, and the immunohistochemical evaluation revealed positivity for TdT, B-lineage markers (CD79a, PAX5, CD19, CD22, and CD10), myeloid marker (MPO), CD34, CD43, FLI-1, and, at least focally, CD33 and CD117 (Figure 2, Figure 3 and Figure 4); the tissue was negative for CD38, intracytoplasmic mu chains, CD13, CD15, monocyte/macrophage lineage markers (CD68, CD11c, CD14, lysozyme, and CD163), T-lineage markers (CD2, CD3, CD4, CD5, CD7, and CD8), Myf4, MND116, and EMA. Proliferation index (Ki67) was 50%. Clonal rearrangement of immunoglobulin heavy chain genes was identified by polymerase chain reaction (PCR) of framework regions (FR) and a dominant peak of 107 bp and 247 bp was seen in FR3 and FR2, respectively. Notably, no residual lymph nodal architecture was observed in all examined tissue sections. Biopsy and bone marrow samples were also screened for the presence of core-binding factor (CBF)-β abnormalities, t(8;21)RUNX1-RUNX1T1, inv(16)CBFB-MYH11, t(15;17)PML-RARA, t(1;19)E2A-PBX1, t(9;22)BCR-ABL p190/p210, t(12;21)TEL-AML1, MLL translocations, and several KMT2A and NUP98 rearrangements [6,7,8]; the internal tandem duplication of FLT3 mutation (FLT3-ITD) and NPM1 mutations were also screened. No genetic mutation was found. According to the WHO criteria [9], a biphenotypic lymphoblastic lymphoma (B/Myeloid) was diagnosed. The staging procedure was completed with thorax–abdomen computerized tomography (CT) scan, whole-body positron emission tomography (PET)-CT scan, and 99Tc-MDP bone scan; no other organ involvement was ascertained. Based upon these findings, in order to use chemotherapy agents effective in both lymphoid and myeloid neoplasms, the patient was started on AIEOP LLA REC 2003 protocol, S2 group, arm B (Table 1). After induction therapy, the clinical and radiological disappearance of the popliteal lesion was appreciated; furthermore, at the end of the chemotherapy blocks, knee MRI and PET-CT confirmed no evidence of disease. The patient has been in continuous complete remission for more than 10 years from stop therapy.

## 4. Discussion

Soft-tissues are uncommon primary sites for NHL, and most of the available information concerns the adult population. In adulthood, PSTL more often affects the extremities, especially the lower limbs, without any sex difference, diffuse large B-cell lymphoma (DLBCL) being the most frequent histological subtype [10].

After literature search, no pediatric review about this topic was found; the discovered case reports are listed in Table 2 [11,12,13,14,15,16,17,18,19,20,21,22] and, including our patient, we identified 13 pediatric PSTL, with a slight predominance of female sex (54%). The median and mean age were 11 and 11.3 years, respectively. In 9 out of 13 children (69%), lymphoma was localized in the extremities, five in the lower limb, two in the upper limb, and two in both upper and lower limbs. Except in our case, all the patients had anaplastic large cell lymphoma (ALCL) as histological subtype (ALK-positive in seven patients). The treatment of choice was chemotherapy alone in 10/13 (77%); chemotherapy was associated with surgery in one case and with autologous stem cell transplantation in another child. In one patient, therapy was not specified [13]. Follow-up was detailed in nine children: seven (78%) achieved complete remission, one relapsed after 6 months from diagnosis and another one died of disease progression less than 2 months after diagnosis. Based on the collected data, pediatric PSTL differs from its adult counterpart in at least two main respects: in adulthood, the prognosis is poor and DLBCL occurs in more than half of all cases [5], while in children, PSTL presents a high rate of survival and ALCL is the predominant histology. In a large international cohort of 420 children and adolescents with ALCL, 48 patients without lymph node involvement at the diagnosis were recognized, of which 40% were soft tissue masses; also in this study, a better prognosis of this rare site of presentation was reported [23].

In a patient presenting with a soft-tissue mass, the most common diagnostic hypothesis is soft-tissue sarcoma, especially desmoid tumors, rhabdomyosarcoma, synovial sarcoma, and Ewing’s sarcoma/pPNET; another differential diagnosis includes neuroblastoma. All these neoplasms belong to the group of small round blue cells tumors, characterized by some degree of cytomorphologic and immunophenotypical overlap [24], but with radically different therapeutic approaches. For example, in lymphoblastic lymphoma (LBL), malignant cells can form rosette-like structures and express CD99 and FLI-1, mimicking Ewing sarcoma [25,26]. The use of a limited immunohistochemical panel is a potential diagnostic pitfall and should be avoided, because a misdiagnosis leads to incorrect treatment and devastating outcomes [27]. To prevent this, small round blue cells tumors should be tested for TdT, CD79a, and CD43; the study of B and T-cell receptor gene rearrangements can also be helpful to distinguish LBL from Ewing sarcoma [28,29].

LBL is the second most common type of NHL in children and adolescents; in around 70–80% of cases, it is of T-lymphoblastic origin and in 20–30%, it arises from B lymphoblasts [30]. Biphenotypic LBL or mixed lineage lymphoma is defined by the presence of tumor cells expressing markers of two lineages, including B-lymphoid, T-lymphoid, and myeloid. It is very rare, even though the frequency is probably underestimated; in a study on 146 pediatric LBLs, an unexpected large number of mixed lineage lymphomas was recognized (7%), B-cell/myeloid differentiation being the most frequent phenotype [31]. For the identification of biphenotypic LBL, an extensive histological evaluation with a systematic staining for all the hematopoietic lineages and molecular genetic analysis should always be performed, as demonstrated in our patient. As regards the treatment, the use of drugs active against lymphoid and myeloid neoplasms seems to be effective in achieving complete remission in the majority of leukemias of ambiguous lineage [32]; this clinical evidence justifies our therapeutic choice, which proved successful.

## 5. Conclusions

Pediatric PSTL is an extremely rare disease, characterized by a favorable prognosis in the majority of patients. In the presence of a soft-tissue mass, histological examination, including an extensive immunohistochemistry panel for lymphoma, and molecular genetic analysis are essential to make a correct diagnosis. In our case, unique because of both onset site (popliteal fossa) and histological characterization (biphenotypic LBL), the choice of a chemotherapy regimen active against both lymphoid and myeloid neoplastic cells showed to be highly effective.

## Figures and Tables

**Table 1:** Therapy courses according to the S2 group/arm B of the AIEOP ALL REC/2003 trial. Our patient received the following chemotherapy: phase IA-IDA, R2 block, R1 block, R2 block, R1 block, R2 block, R1 block, maintenance.

Drug	Dose
**Phase IA-IDA days 1–30**	
Prednisone	60 mg/m^2^ orally days 1–30 °
Vincristine	1.5 mg/m^2^ IV days 1, 8, 15, 22
Idarubicin	6 mg/m^2^ IV days 1, 8, 15, 22
L-Asparaginase	10,000 UI/m^2^ IM days 5, 8, 11, 14, 17, 20, 23, 26
**R2 block days 1–6**	
Dexamethasone	20 mg/m^2^ orally days 1–5
6-Thioguanine	100 mg/m^2^ orally days 1–5
Vindesine	3 mg/m^2^ IV day 1
High-dose Methotrexate	1 g/m^2^ IV (36 h) day 1
Ifosfamide	400 mg/m^2^ IV (1 h) days 1–5
Daunorubicin	35 mg/m^2^ IV (24 h) day 5
L-Asparaginase	10,000 UI/m^2^ IM day 6
**R1 block days 1–6**	
Dexamethasone	20 mg/m^2^ orally days 1–5
6-Mercaptopurine	100 mg/m^2^ orally days 1–5
Vincristine	1.5 mg/m^2^ IV days 1 and 6
High-dose Methotrexate	1 g/m^2^ IV (36 h) day 1
High-dose Cytarabine	2 g/m^2^ IV (3 h), two doses 12 h apart, day 5
L-Asparaginase	10,000 UI/m^2^ IM day 6
Maintenance treatment *	
Methotrexate	20 mg/m^2^/week orally
6-Mercaptopurine	50 mg/m^2^/day orally

° with a scalar dose from day 21. * The total duration of therapy including maintenance was 24 months. IV: intravenous; IM: intramuscular.

**Table 2:** Pediatric patients affected by PSTL reported in the literature.

Reference	Age/Sex	Primary Site	Histology	Therapy	Follow-Up
Winter et al. (1991) [11]	8 y/M	Buttock	ALCL (ALK ND)	CT	CR
Evans et al. (1993) [12]	10 y/F	Left wrist and forearm (flexor muscle)	ALCL (ALK ND)	Surg + CT	Relapse, 6 m
Chew et al. (1999) [13]	16 y/F	Thigh (sartorius muscle)	ALCL (ALK ND)	NS	NS
Ishii et al. (2000) [14]	11 y/F	Right arm	ALCL (ALK -)	CT + aHSCT	CR, 2 y
Menon et al. (2001) [15]	10 y/F	Left thigh (rectus femoris muscle), left arm and chest wall	ALCL (ALK ND)	CT	CR
Driss et al. (2009) [16]	8 y/M	Right buttock	ALCL (ALK +)	CT	CR
Wu et al. (2009) [17]	14 y/M	Left sacrospinalis, lumbar and femoral muscles	ALCL (ALK +)	CT	Dead, 2 m
Rekhi et al. (2010) [18]	9 y/F	Right pectoralis major muscle, right axilla and lateral chest wall	ALCL (ALK +)	CT	NS
Gaiser et al. (2012) [19]	10 y/M	Left biceps femoris muscle	ALCL (ALK +)	CT	NS
Kounami et al. (2012) [20]	14 y/F	Left major psoas and iliopsoas muscles	ALCL (ALK +)	CT	CR, 4 y
Pasricha et al. (2013) [21]	14 y/F	Right biceps brachii and right gluteal muscles, left arm, right thigh, left chest wall	ALCL (ALK +)	CT	CR, 7 m
Gupta et al. (2022) [22]	12 y/M	Left thigh and chest wall	ALCL (ALK +)	CT	NS
Our case	11 y/M	Right popliteal fossa	BLLy	CT	CR, 11 y

**Abbreviations:** y: years; M: male; ALCL: anaplastic large cell lymphoma; ND: not done; CT: chemotherapy; CR: complete remission; F: female; Surg: surgery; m: months; NS: not specified; aHSCT: autologous hematopoietic stem cell transplantation; BLLy: biphenotypic lymphoblastic lymphoma.

## Data Availability

The data that support the findings of this study are available on request from the corresponding author. The data are not publicly available due to privacy or ethical restrictions.
